# Assessing attachment representations among adoptees during middle childhood and adolescence with the Friend and Family Interview (FFI): clinical and research perspectives

**DOI:** 10.3389/fpsyg.2014.01114

**Published:** 2014-10-01

**Authors:** Cecilia Serena Pace

**Affiliations:** Department of Educational Science, University of GenoaGenoa, Italy

**Keywords:** attachment, adoption, friend and family interview, middle childhood, adolescence, Internal Working Models (IWMs)

## Adoption and attachment theory beyond infancy and early childhood

Three trends of attachment research could be identified in the adoption field. The first was focused on the comparisons between attachment patterns of adopted children and those of their non-adopted peers. It indicates a higher probability of insecure and disorganized patterns among the adoptees (Van den Dries et al., 2009). The second highlighted a significant intergenerational concordance of attachment Internal Working Models (IWMs) between adoptive parents and their adopted children (Steele et al., 2003; Barone and Lionetti, 2012). The third revealed—through longitudinal design—the increasing security of adopted children which is positively affected by the parental attachment security, especially maternal one (Beijersbergen et al., 2012). This trend suggests that adopted children have the ability to generate new relationships of attachment with adoptive parents, although the impact of their previous adverse experiences has not completely disappeared (Steele et al., 2008; Pace and Zavattini, 2011).

Recently, a new research trend has been emerging: the assessment of adoptees' IWMs in the course of middle childhood and adolescence, not exclusively focused on their relationship with parents. Many important changes happen at the emotional, cognitive and behavioral level during these stages which are related to attachment systems (Steele and Steele, 2005; Allen, 2008). Adolescents develop the meta-cognitive skills to check their mental states, as well as the ability to recognize positive or negative aspects in their relationships with parents. Moreover, they seek greater independence, greater autonomy and differentiation from their primary caregivers. Relationships with other people outside the family (friends, etc.) become much more important than they had ever been before (Allen, 2008). Due to these changes, middle childhood and adolescence could be considered periods of potential transformations at level of attachment representations. Therefore, they become relevant in the study of adoptees behavior (Palacios and Brodzinsky, 2010).

## The friend and family interview (FFI)

The evaluation of attachment representations during middle childhood and adolescence is done using narrative tools, such as story-completion tasks or interviews (e.g., Attachment Interview for Childhood and Adolescence; Riva Crugnola et al., 2009). These tools allow us to go more in-depth into the adolescent's story and obtain a more extensive study of the richness of his or her experiences with regard to attachment relationships. The *Friends and Family Interview* (FFI, Steele and Steele, 2005; Steele et al., 2009) is an increasingly used narrative method with adopted samples (see below). The FFI is a semi-structured interview, informed by but distinct from the *Adult Attachment Interview* (AAI, Main et al., 2008) which is considered the “golden standard” method to assess attachment states of mind of adults. As the AAI, the FFI is aimed at capturing the individual differences of attachment representations (*secure, dismissing, preoccupied*, and *disorganized*). It focuses on the *coherence* of the narrative leaning heavily toward Grice's (1975) maxims of “good conversation,” i.e., being truthful, relevant, economical and conventionally polite. Therefore, the core of the coding system is based on the “style of discourse,” rather then its—positive or negative—content. Two main differences have emerged between the FFI and the AAI: (1) the questions of the FFI focus, in turn, on self, peers (best friend), siblings and parents, not exclusively on the interviewee-parents relationships (like the AAI), (2) the FFI is a way of systematically inquiring about the young person's view—supported by episodes—of the complex and often conflicting emotions arising in one's closest relationships, rather than comparing semantic (adjectives) and episodic memory (episodes) of the past experiences with attachment figures (like the AAI). Although the AAI was developed in the area of development research, it has revealed precious clinical applications, i.e., facilitating therapeutic alliance, assessing therapeutic outcomes, indentifying defensive processes, etc. (Steele and Steele, 2008). My question is whether the FFI could possibly have relevant clinical applications (as the AAI had) specifically in the adoption context. In addressing this question, I will briefly review literature that analyzed attachment models of adopted children and adolescents with the FFI, discuss questions left unanswered by the current literature, and set the path for future clinical applications and research concerns.

## Capturing attachment representations among adoptees by the FFI: research to date

I identified three research groups who analyzed the attachment representations of adoptees from middle childhood to adolescence using the FFI (Steele et al., 2009). In Table 1 see the distribution of attachment classifications among adoption studies using the FFI.

**Table 1:** **The distribution of attachment classifications among adoption studies using the FFI**.

**Studies with FFI**	***N***	**Type of adoption**	**Age**	**Non-adopted controls**	**Attachment classifications**			
					**Secure (%)**	**Dismissing (%)**	**Preoccupied (%)**	**Disorganized (%)**
Abrines et al., 2012	58	Only international	7–8 (*M* = 7.48)	no	60	26	12	2
Barcons et al., 2012	116	Only international	8–11 (*M* = 8.92)	no	60.3	25	12.9	1.7
Barcons et al., 2014	168	Only international	7–11 (*M* = 8.33)	no	58.9	25	13.1	3
Stievenart et al., 2012	78	Only Domestic (Belgian and Romanian)	10–16 (*M* = 13.3 and 12.9)	no	/	/	/	/
Groza et al., 2012	39	Only domestic (Romanian)	11–16 (*M* = 13.1)	no	51.3	48.7		0
Escobar and Santelices, 2013	25	Only domestic (Chilean)	11–18 (*M* = 12.9)	yes	32	52	16	0
Pace et al., 2013a	22	International and domestic (Italian)	12–16 (*M* = 14)	no	64	27	9	0
Pace et al., 2013b	16	International and domestic (Italian)	12–14 (*M* = 13.2)	no	56	31	13	0

The first research group focused on the correlations between attachment security/insecurity and several features of children's psychological functioning. Abrines et al. (2012) found that internationally adopted children with a secure attachment showed significantly less attention problems and a trend toward less hyperactivity. Barcons et al. (2012) highlighted a significantly better interpersonal and parental relationships among adoptees classified as secure by the FFI. Lastly, Barcons et al. (2014) indicated that a secure attachment representation facilitates the development of adaptive skills (adaptability, social skills and leadership skills) of adoptees. From the second group, the Attachment Adoption Adolescents Research Network (AAARN), Stievenart et al. (2012) demonstrated—through a psychometric study—the measurement invariance of the FFI from Belgian and Romanian adopted adolescents. This study showed that the FFI coherence was similar across the two samples and correlated with the attachment categories. Among domestically adopted Romanian children, Groza et al. (2012) found that little more than the half had secure attachment representations and none showed disorganized ones. Lastly, Escobar and Santelices (2013) pointed out that in Chile nationally adopted adolescents showed more insecure attachment—mostly dismissing—than their non-adopted peers. The third research group found a significant concordance between secure/insecure attachment classifications of mothers by the AAI and late-adopted adolescents by the FFI (Pace et al., 2013a). In addition, Pace et al. (2013b) highlighted a significant change of attachment classifications. Growing from beginning of adoption to adolescence children moved from insecurity toward security through a long-term longitudinal research design.

## Clinical and research issues

### Reflective functioning (RF)

The link between poor mentalization skills—often operationalized in terms of RF—and severe difficulties in the early parent-child relationships is widely acknowledged. Much less it is known regarding mentalization skills of adoptees, even if low RF was strongly associated with early relational traumas which are common in their pre-adoption lives. On one hand, traumatic experiences—such as abuse and maltreatment perpetuated by original attachment figure during infancy and childhood—can inhibit the development of RF: thinking of the other's mind for traumatized becomes a very dangerous experience. On the other hand, high RF in individuals who have experienced early adversities seems to represent a resilience factor. It can reduce both intergenerational transmission of insecurity and the probability of onset of borderline personality disorder (Fonagy and Bateman, 2006). One of the merit of the FFI's coding system is that RF is operationalized across three sub-domains: (1) *developmental perspective* that represents the interviewee's capacity to contrast his/her current thoughts and feelings concerning important relationships or his/her self-view with past attitudes; (2) *theory of mind*, intended as the ability to assume the mental or emotional perspective of another person; (3) *diversity of feelings*, defined as the ability to show an understanding of diverse (negative and positive) feelings being present in significant relationships involving self and other people (Kriss et al., 2012). Despite the importance of RF, none of the above-mentioned adoption studies reported data on it. I suggest that the assessment of the RF through the FFI of adopted children and adolescents could be relevant: (1) from a clinical perspective, because it can help adoption workers to individuate a vulnerable factor (low RF) that needs to be enhanced; (2) from a research perspective, in order to analyze the precursors, concomitants and sequelae of RF of adoptees.

### Disorganization

Disorganized IWMs are considered connected both with early maltreatments and later long-term psychopathological outcomes (Steele and Steele, 2008). Studies on late-adopted children (aged 3–8) showed—in line with their early adverse experiences—a consistent presence of disorganization among them (over 30%) assessed through attachment completion tasks (Steele et al., 2003, 2008; Barone and Lionetti, 2012; Pace et al., 2012, 2013c). Surprisingly, disorganized IWMs appear rather under-represented in the adoption studies with FFI showing a percentage from 0 to 3%. A lower result compared to non-clinical adolescent populations assessed by the AAI (around 11%, Bakermans-Kranenburg and Van Ijzendoorn, 2009). I suppose that it would be rather difficult to think that disorganized representations, revealed among adopted children at previous stages, tend to “disappear” at later stages. The FFI coding system may not capture subtle cues of disorganization at a narrative level as both attachment completion task (e.g., catastrophic fantasy, bizarre/atypical material, child parents/controls, extreme aggression, etc.) and the AAI (e.g., lapses of monitoring of reasoning/discourse about loss/trauma) can do. However, I suggest the hypothesis that the FFI should include the evaluation of two non-verbal codes which could be connected with disorganized classifications: (1) distress and fear, (2) frustration and anger. These non-verbal codes capture specific signs of distress such as freezing behavior, stereotypic movements, verbal and non-verbal aggression toward the interviewer, etc. which are usually considered indexes of disorganization (Steele et al., 2009). Up today, none of the FFI-related adoption studies have explicitly taken into consideration these codes. I suppose that the inclusion of the non-verbal codes could be useful with adoptees for two reasons: (1) from a clinical perspective, because it can help clinicians to understand adoptee's level of integration/non integration among behavioral and somatic vs. cognitive and affective expressions of self (e.g., an adolescent describing very violent fights with his father, but these are referred with a cool detachment), (2) from a research perspective, to explore whether they were correlated with disorganized classifications and, in turn, whether disorganization develops throughout longitudinal research design.

### Reflections on some FFI questions

In the FFI the child/adolescent is asked to think back to his earliest memory of separation from caregivers, first in terms of his own behavior, thoughts, and feelings. Subsequently in terms of how he imagines his caregivers might have felt at the time (n. 21). Then he/she is asked to speak about his/her relationships with his/her brother and sister (n. 22).

I argue that these specific questions could often assume a unique meaning for adoptees. Indeed late-adopted children often had spent long time with their biological parents and siblings and they have lively and clear memories of them. For examples, it happen that adopted speaker asks to the interviewer “*Separation from who? My biological parents or my adoptive parents?*,” or he/she can say “*I have not sibling now here. But I had four older brothers in Brazil, where I was born. Would you like that I speak about them?*”. I suggest that leaving these questions open (without any limitation like: “*I want you to speak about separation from your adoptive parents/your living-with sibling*”) could provide interesting and relevant information about adopted population. From a clinical perspective, the answers to these questions can assume a great importance during treatment of adoptive families, because they help to understand how the process of integration of past and present representations has been working in the inner world of the adoptees. From a research perspective, maybe it could be useful to capture this information in specific codes for adopted samples and explore whether they are connected with individual and familiar factors.

## Conclusions and recommendations

In conclusion, the FFI is a novel method developed to assess the IWMs of individuals that range from 9 to 17 years of age and it was often used with adopted samples. A small but emerging adoption literature highlighted that adoptees with secure attachment representations measured by the FFI showed significantly less psychological problems and more competences compared to insecure ones. In addition significant associations between adoptees secure IWMs and maternal secure attachment states of mind were detected. However, the FFI, and its coding system, shows some limits—such as a lower skill of detecting disorganization compared with other measures—that need to be taken into consideration. Future research needs to consider not only the global attachment classifications (secure, dismissing, preoccupied and disorganized), but also other dimensions coded through the FFI (coherence, reflective function, evidence of secure base, etc.) that could offer more relevant information about the inner world of the adoptees. In addition, the FFI could be an attachment measurement (as yet it happened with the AAI) useful to yield valuable insights into the emotional and relational difficulties of adopted children/adolescents, facilitating case formulation and treatment planning of post-adoption services.

### Conflict of interest statement

The author declares that the research was conducted in the absence of any commercial or financial relationships that could be construed as a potential conflict of interest.
